# *X-ray Spectral Imaging Program: XSIP*

**DOI:** 10.1107/S1600577520010838

**Published:** 2020-09-14

**Authors:** Peng Qi, Nazanin Samadi, Dean Chapman

**Affiliations:** a Division of Biomedical Engineering, University of Saskatchewan, Saskatoon, Canada; b Swiss Light Source, Paul Scherrer Institute, 5232 Villigen, Switzerland; c Anatomy, Physiology and Pharmacology, University of Saskatchewan, Saskatoon, Canada

**Keywords:** wide-field energy-dispersive XAS, spectral KES, Python

## Abstract

Spectral *K*-edge subtraction imaging and wide-field energy-dispersive X-ray absorption spectroscopy imaging are novel, related, synchrotron imaging techniques for element absorption contrast imaging and element speciation imaging, respectively. These two techniques serve different goals but share the same X-ray optics principles with a bent Laue type monochromator and the same data processing algorithms. As there is a growing interest to implement these novel techniques in synchrotron facilities, Python-based software has been developed to automate the data processing procedures for both techniques. In this paper, the concept of the essential data processing algorithms are explained, the workflow of the software is described, and the main features and some related utilities are introduced.

## Abstract 2

An open source Python software package, developed for data analysis for spectral *K*-edge subtraction imaging and wide-field energy-dispersive X-ray absorption spectroscopy imaging, is reported.

## 1. Introduction   

Bent Laue or transmission type silicon crystals have been widely used as monochromators in synchrotron X-ray studies, including *K*-edge subtraction imaging (KES) (Elleaume *et al.*, 1999 ▸) and energy-dispersive X-ray absorption spectroscopy (EDXAS) (Pascarelli *et al.*, 2016 ▸). KES and EDXAS are X-ray techniques that serve different research purposes and have different experimental procedures and data analysis. Recently, the developments of a spectral KES imaging technique and a wide-field EDXAS imaging technique blur the boundary between these two techniques as they share the same principle, similar instrumentation and similar data analysis method. The differences between the two imaging techniques are that spectral KES is meant for studying the absorption contrast between an element and its matrix at relatively high energies (*e.g.* >20 keV), while the wide-field EDXAS is for investigating the local environment and/or the speciation of an element at lower energies (*e.g.* <15 keV) where there is informative absorption structure in the near-edge region of the element. Spectral KES and wide-field EDXAS imaging will be referred to as ‘spectral imaging’ for simplicity, because of the commonality of the two imaging techniques.

Both spectral KES and wide-field EDXAS are enabled by a bent Laue monochromator with excellent energy-dispersive properties (Zhu *et al.*, 2014 ▸; Qi *et al.*, 2019 ▸). This improved energy resolution, especially in the case of KES, has resulted in the elimination of a splitter to separate the X-rays into above and below the *K*-edge of the contrast element beams, resulting in a simpler, easier-to-align and operate imaging system. This ease of use, however, has resulted in a more complex image analysis requirement. The program described here was developed to take advantage of the improvement in energy resolution for KES and XAS applications.

As there is a growing interest in implementing these novel imaging techniques in facilities worldwide, a user-friendly Python-based software, the *X-ray Spectral Imaging Program* (*XSIP*), was developed for spectral imaging data analysis.

## 2. Theory   

In conventional KES, two X-ray absorption images are collected at energies below and above the edge (usually the *K*-edge) energy of the contrast element. A negative logarithm subtraction is performed between the two absorption images so that the absorption jump by the contrast agent at the edge energy is prominent from the background absorption. In other words, the two measurements made allow for solving for two materials given the attenuation properties of the two materials.

The spectral imaging system prepares a focused line X-ray beam that has a small focal size and a good energy dispersion property in the focusing dimension (*e.g.* the vertical hereafter), while the other dimension (*e.g.* the horizontal) of the beam forms a line at the focus where the object to be imaged is located.

According to Bragg’s law, the diffraction angles and the energies of the X-rays in the focused beam are correlated. When an area detector is hit by the beam at a certain distance, the angle–energy correlation then results in a position–energy correlation on the detector in the vertical dimension. Having the monochromator match the so-called ‘magic condition’ (Martinson *et al.*, 2015 ▸) is critical for achieving a small focal size and good energy resolution.

When a sample is placed at the focal line, the X-rays at all energies in the beam are attenuated by the sample, and the transmitted X-rays propagate to the detector. The pixels on the detector capture the transmitted X-ray signal, and the vertical positions of these pixels on the detector provide the energy information of the captured X-ray photons. Therefore, an attenuation spectrum for every horizontal position of the sample is captured. Because the shape of the beam at the focus is a line beam, the contrast materials are quantified in the horizontal dimension and by scanning the sample in the vertical dimension a 2D distribution of the contrast materials is obtained. Similarly, a rotation of the sample can be used to generate a computed tomography (CT) image of the materials. Detailed descriptions of the system are given by Zhu *et al.* (2014 ▸) and Qi *et al.* (2019 ▸).

To solve for the quantities of the subject materials in the beam, the two-equation problem in KES becomes an *n*-equation problem or a curve-fitting problem in the spectral KES case. Regardless of the number of materials, as long as it is less than *n*, the equation system is over-defined and a least-squares fit algorithm is used for the best solution of the quantities.

Reference mass attenuation spectra [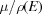 as a function of energy *E*] of the materials are needed for the least-squares fit calculation and proper extraction of quantifiable projected density values (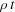, the mass density and thickness product). For spectral KES which is suitable for high energies, the references are calculated with built-in functions (including absorption, elastic scattering and inelastic scattering) in the program, because of the simplicity of the near-edge absorption spectrum of high-atomic-number elements. For wide-field EDXAS, the reference data can be external measurements by dedicated XAS beamlines or internal using the spectral imaging system. Reference data measured by the system are preferred as they account for the monochromator energy resolution and energy calibration.

## 3. Program workflow   

**Figure 1:**
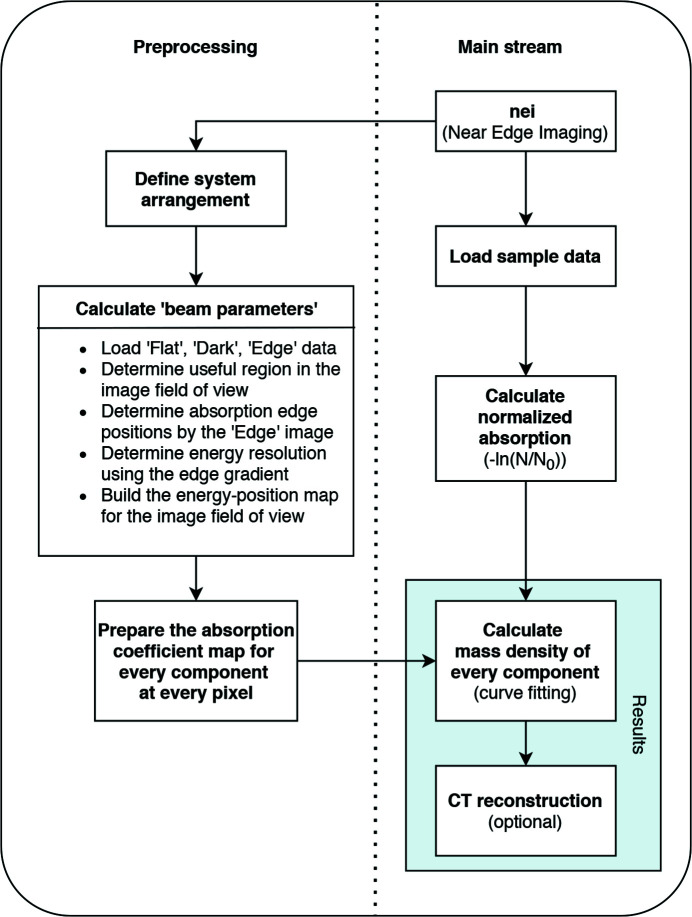
Overview of the *XSIP* workflow. The program starts by calling the ‘nei’ function with a number of user-defined parameters and ends with the outputs in forms of images and a PKL format file containing intermediate results during the data analysis.

The core algorithm of the spectral imaging data analysis is to fit a series of reference spectra to the measured spectrum at every location on the sample. The workflow of the program includes defining system settings and loading the data, preprocessing, curve fitting for near-edge absorption data and CT reconstruction if needed. A flowchart of the program is shown in Fig. 1 ▸.

### 3.1. System settings and data loading   

Some parameters of the imaging system should be defined prior to the data analysis. The critical ones are the Laue crystal asymmetry angle (Zhu *et al.*, 2014 ▸), reflection reciprocal indices, the contrast element *K*-edge X-ray energy, the effective pixel size of the detector and the distance between the focus of the diffracted beam and the detector. This information is essential for analysis. These parameters can be input either with an 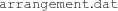 file, which will then be read by the program, or with manual input in a prepared pop-up window when the 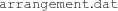 file is unavailable or not preferred. An example of the arrangement file is given in the GitHub repository referred to in the *Summary* section[Sec sec5].

For proper data analysis, the images from the detector need to be corrected for dark current and detector response (flat-field normalization). Correction images in the form of beam off images, *i.e.* ‘dark’ images, and beam on with no sample images, *i.e.* ‘flat’ images, are required. A number of dark images and flat images (ten images for each by default) are read in and averaged. A number of ‘edge’ images are read in for the energy calibration. These images are taken with an elemental form of the contrast material in the beam with no sample present. Data images with the sample or ‘tomo’ images are read in and are grouped by slice if a multi-slice CT scan was done.

### 3.2. Prepocessing   

#### 3.2.1. Dark and flat normalization   

The averaged dark images and averaged flat images are used to normalize the absorption of X-rays by the sample, as shown in equation (1),
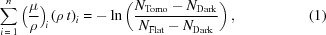
where 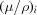 is the energy-dependent mass attenuation coefficient of component *i* inside the sample, 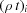 is the projected density of component *i* contributing to the attenuation and *N* is the measured photon count for dark, flat and tomo images as marked by the subscriptions, respectively.

The flat image is also used for determining the useful field of the beam. Due to the nature of the synchrotron source X-rays, the beam intensity profile is a near Gaussian distribution in the vertical dimension. Also, the detector may have spatial variations in response that need to be accounted for in determining the useful beam region.

#### 3.2.2. *K*-edge position and energy distribution on the detector   

**Figure 2:**
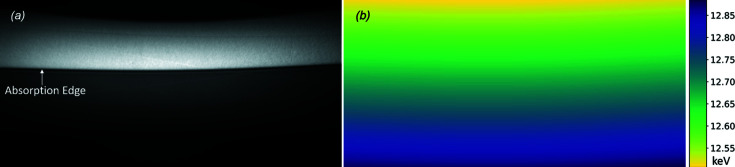
An example edge image. (*a*) A 0.2 mm selenium film is used as the edge reference material. The arrow indicates the absorption edge at 12.658 keV. Data collected on 10 September 2015. (*b*) Energy mapping in the total image field of view.

To determine the energy distribution information on the detector, the element under investigation in the form of a solid film or solution was placed in the beam for collecting the reference edge images. An example of the edge image is shown in Fig. 2 ▸(*a*). There is a noticeable curvature in the beam due to non-uniform bending of the monochromator. At the sample, which is less than one half of the distance between the monochromator and the detector, the curvature is not as large and not noticeable given the vertical beam size at that location. More uniform bending of the monochromator can eliminate this effect.

Because of the absorption jump at the edge energy, the edge image is darker on the high-energy side and brighter on the lower-energy side. In theory, the border of the two regions is a sharp transition. The transition takes place in a small energy range (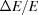 ≃ 10^−4^) because of the core-hole lifetime and the monochromator energy resolution. Derivatives of all the columns of the edge image are taken and the peak index of the derivative curve sets the edge energy.

**Figure 3:**
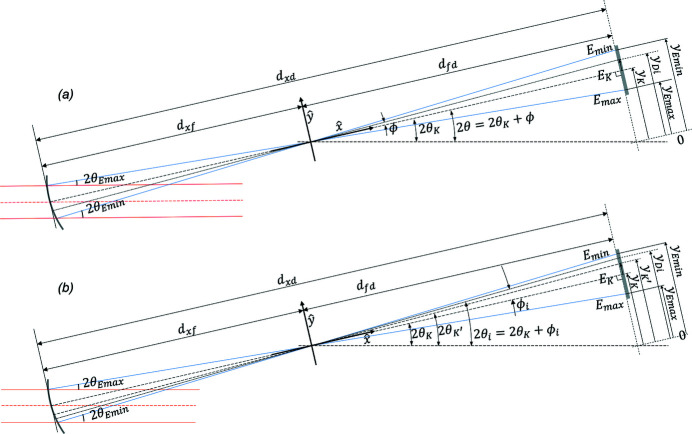
The geometry for relating the diffraction angles and the pixel positions on the detector with (*a*) one reference energy and (*b*) two reference energies. The *K*-edge energy, the monochromator focal distance, the focus-to-detector distance, the Bragg plane indices and the crystal asymmetry angle are the determining factors.

The energy-position information for every pixel of the detector in the diffraction plane (typically the vertical plane) can then be determined using Fig. 3 ▸(*a*). The relation between energy and detector pixel position is given by
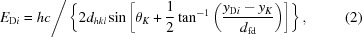
where 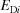 is the energy at the *i*th pixel of the detector, *h* is the Planck constant, *c* is the speed of light, 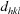 is the lattice spacing of the crystal Bragg planes, 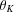 is the Bragg diffraction angle of the X-rays at the *K*-edge energy, 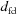 is the focus-to-detector distance, 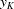 is the detector pixel location of the *K*-edge energy and 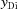 is the *i*th detector pixel location. An example of the energy mapping in the detector field of view is shown in Fig. 2 ▸(*b*).

The energy mapping can be performed in another way which is more accurate but requires reference material with identifiable absorption features at two energies. For example, the near-edge absorption spectrum of selenate [in pH 7.4 bicine buffer (C_6_H_13_NO_4_)] has two absorption peaks at 12.667 keV and 12.681 keV. With the pixel positions and the energies of the two peaks, the energy distribution on the detector can be determined as shown in Fig. 3 ▸(*b*) and equation (2), where 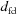 can be substituted with
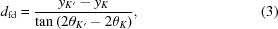
where 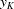 and 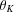 are the pixel position and the Bragg diffraction angle corresponding to the X-rays with energies at the first absorption peak, 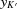 and 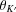 are the pixel position and the Bragg diffraction angle corresponding to the X-rays with energies at the second absorption peak.

The edge image is also useful for determining the energy resolution of the system. Specifically, the width of the absorption edge is calculated as the full width at half-maximum (FWHM) of the derivative of the edge absorption spectrum. This value, converted to a Gaussian width, is used with a Gaussian filter to blur an externally measured reference by dedicated beamline to match the measured resolution.

### 3.3. X-ray absorption near-edge curve fitting   

Reference mass attenuation spectra of the materials are needed for the calculation, which can be generated internally, externally or measured as described before.

**Figure 4:**
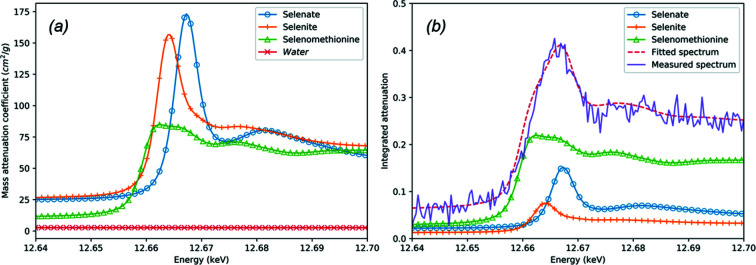
An example of spectra curve fitting. (*a*) The mass attenuation coefficient of reference materials. (*b*) Measured absorption spectrum and fitted absorption spectrum with reference materials. The reference spectra are also shown in the figure with their fitted weight. The attenuation by *Water* is removed from the total attenuation for better demonstration.

Every column of a projection image contains the spectrum of transmitted X-rays for the corresponding location of the sample. The negative log of these transmission values are then generated to create effective ‘
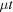
’ values as a function of energy. With a list of reference mass attenuation spectra for all possible constituents, a least-squares fitting is used to solve for the projected density of all constituents that best fit the effective ‘
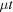
’ values (Zhu *et al.*, 2014 ▸; Qi *et al.*, 2019 ▸). This is described in more detail by Qi *et al.* (2019 ▸) in the *Analysis Method* subsection in their *Methods* section. An example result for the curve fitting with least-squares approach is shown in Fig. 4 ▸. In the case that some constituents are not present in the sample, the linear coefficient for the projected density is zero, theoretically. With an extensive reference library, knowledge of possible constituents in the sample prior to the data processing is not required. However, over the energy range measured, the mass absorption coefficient needs to be distinctively different as a function of energy.

**Figure 5:**
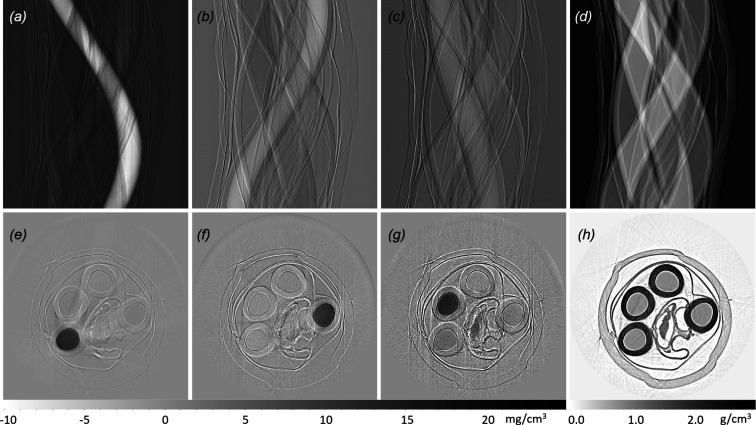
Sinograms of the distribution of (*a*) selenate, (*b*) selenite, (*c*) selenomethionine and (*d*) water. The CT reconstruction of the density distributions of (*e*) selenate, (*f*) selenite, (*g*) selenomethionine and (*h*) water. Every Se compound solution is prepared with 100 m*M* concentration. 7 mg cm^−3^ selenomethionine was detected in the seedpod of *Astragalus bisulcatus*, which is placed in the center of the sample holder. The gray scale bar on the bottom left is shown for the corresponding Se compounds concentrations in (*e*), (*f*) and (*g*), and the gray scale bar on the bottom right for (*h*).

Therefore, a horizontal projected concentration of every reference component is acquired from one sample position. With a series of projections by scanning the sample vertically, a 2D distribution of projected concentrations is determined. With a series of projections by rotating the sample, a sinogram of every species can be acquired, which, when constructed, will give density values of various components. An example is shown in Fig. 5 ▸.

In terms of programming for the least-squares fit calculation, the ‘torch.tensor’ object from the ‘PyTorch’ package (Paszke *et al.*, 2019 ▸) is preferred to the more popular ‘numpy.array’ (Oliphant, 2006 ▸). Although the PyTorch package is dedicated to machine learning tasks, the program takes advantage of its fast calculations for large multiple dimension matrices. Roughly, it reduces by two thirds the time used for the least-squares fit with ‘numpy.array’.

The previous examples show the results of wide-field EDXAS imaging. Spectral KES data analysis can be performed the same way. The only difference is that the provided reference material list is somewhat limited to the contrast element and a matrix material, such as water. The lack of edge structure prevents solving for species of the contrast element and the lack of strong energy dependence of the matrix material will prevent solving for more than one component.

## 4. Features   

### 4.1. Main task   

Sharing the same principle theory and system design, experiment data for both spectral KES and wide-field EDXAS can be processed by the *XSIP* software.

With the input being the dark images, the flat images, the edge images and the tomo images (the real data of the sample), the outputs of the program are individual projected densities of the reference materials. Those reference materials not present in the sample will simply result in projected densities at or near zero.

### 4.2. Result storage   

During data processing, some important mid-way results have been collected and saved together with the final result in a specified destination. Some examples are the energy mapping in the beam, the useful beam region by a threshold to the intensity of the flat image, the reference materials spectra in-use and the attenuation data corrected by the flat and dark images. These mid-way results are useful when in-depth evaluation of the data is necessary.

All results are saved in a PKL format file (Van Rossum & Drake, 2009 ▸) for future access by the program. The final results are also saved in the TIF format images for visualization.

The system settings and the main function parameters used for the analysis are saved in a TXT file as a record for future reference.

### 4.3. Utilities   

#### 4.3.1. CT reconstruction   

A CT reconstruction tool is provided in the program. It is a wrapper of the ‘iradon’ function from the ‘scikit-image’ Python package (Van Der Walt *et al.*, 2014 ▸), which applies a filtered back projection algorithm (Kak & Slaney, 1988 ▸). Options for desired reconstruction filters provided by ‘scikit-image’ are available. In addition, a function for finding the rotation center index automatically in the CT projections is also provided.

A typical CT scan with the spectral imaging system can take about 10 min (the time varies depending on the exposure time and number of projections per CT slice). During the scanning, the incident photon flux decreases because of the decay of the electron beam in the storage ring, which has an adverse influence on the data analysis. Therefore, preferred CT projection data will have an area on the left and right side of the field of view that is empty during the scan (no sample is presented in this area). In this case, the changes of the signals in the empty areas are only due to the electron beam decay and can be used to fix this issue.

#### 4.3.2. Magic condition related calculators   

The core principle that enables the small focal size and good energy resolution of the spectral imaging system is the magic condition (Martinson *et al.*, 2015 ▸). Thus, some magic condition related calculators are provided in the program for designing and evaluating magic condition monochromators.

(i) *Variables for designing a magic condition monochromator.* The variables required to designed a magic condition bent Laue monochromator are: χ (asymmetry angle), θ (center ray Bragg angle), *R* (crystal bending radius), *D* (X-ray source distance) and ν (Poisson ratio of the crystal, which is assumed to be uniform in the diffraction plane of the crystal). This calculator (‘xsip.math_physics.magic_condition’) is used for calculating one variable to match the magic condition while the other variables are assumed to have known values.

(ii) *Metric for magic condition evaluation.* Due to practical limitations, such as the cost of customized crystals, the limited space of a beamline or using the same monochromator for different energy applications, sometimes the monochromator is seldom at the perfect magic condition. In this scenario, an evaluation for the ‘distance’ to the magic condition is necessary. The quasi-monochromatic beam width (‘xsip.math_physics.quasi_mono_beam_width’) is a useful metric for this purpose (Qi *et al.*, 2020 ▸). It measures the width of an X-ray beam parallel to the diffracted center X-ray, which is zero when the monochromator matches the magic condition. It can be directly related to both the broadened focal size and the blurred energy resolution.

#### 4.3.3. Calculator for energy resolution   

A good energy resolution is important for XAS studies. This function (‘xsip.math_physics.relative_energy_resolution’) calculates the theoretical energy resolution of the magic condition monochromator. The algorithm used in this calculator is more accurate than the classic way which sums all contributions in quadrature using a Gaussian distribution model. It calculates the mutual effect of the contributions of crystal lattice spacing variation and the finite source distance. Details are given by Qi *et al.* (2020 ▸).

### 4.4. A graphic user interface   

**Figure 6:**
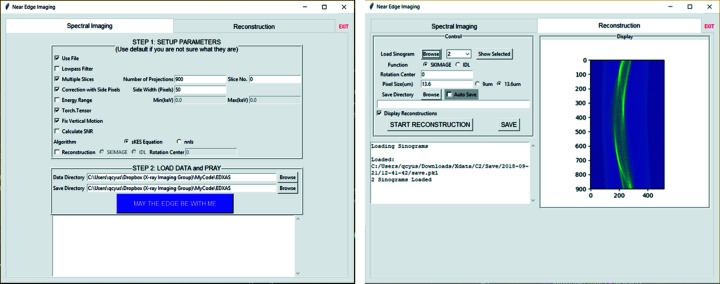
Screenshots of the XSIP GUI. (*a*) The ‘Spectral Imaging’ panel contains the main data analysis pipeline and several parameters to tune for best result. (*b*) The ‘Reconstruction’ panel contains the reconstruction tool and a display canvas for the sinogram and reconstruction results.

A graphic user interface (GUI) is available in addition to using command line functions. As shown in Fig. 6 ▸, there are two panels contained in the GUI. The ‘Spectral Imaging’ panel contains the main data analysis pipeline and options to tune several parameters to optimize the data analysis. The ‘Reconstruction’ panel contains available reconstruction tools and a display canvas for sinogram and reconstruction results. The GUI was built with the ‘tkinter’ package built-in with Python 3.5+.

## 5. Summary   

The *X-ray Spectral Imaging Program* (*XSIP*) is built specifically for the spectral KES and wide-field EDXAS imaging techniques, which have been deployed at the Canadian Light Source (CLS) BMIT-BM beamline. The program automates the analysis of the data generated from the two imaging methods and provides the flexibility of several parameters for optimizing the analysis. Quantified projected density distributions of the reference materials are the principle outputs of *XSIP*. In the case of computed tomography, the sinograms can be optionally constructed. A GUI is provided for the essential functions and result visualization.

The software evolved as the system became more useful for biomedical research. The experience gained while the system was used at the CLS BMIT-BM beamline (Bassey *et al.*, 2015 ▸, 2016 ▸; Panahifar *et al.*, 2016*a* ▸,*b* ▸, 2019 ▸; Deman *et al.*, 2017 ▸) led to the current *XSIP* program.

It is relatively simple to set up and operate a spectral KES-based imaging system; the complexity is in the data analysis due to the multiple energies of the imaging beam. This type of spectral imaging system is being considered by other bio­medical imaging programs at other facilities. The intent of this program is to remove some of the data analysis complexity, and thus enable the system’s possible use elsewhere.

*XSIP* is an open access software developed with Python 3.6. It is accessible at https://github.com/darwinqii/XSIP along with detailed documentation for installation and user guide for the functions introduced in this work. The program is compatible on Windows (tested on Windows 10) and Linux (tested on Ubuntu 18.04.3 LTS and CentOS 7) platforms. MacOS has not been tested.
